# Exploring the Impact of Genetics in a Large Cohort of Moebius Patients by Trio Whole Exome Sequencing

**DOI:** 10.3390/genes15080971

**Published:** 2024-07-23

**Authors:** Giada Moresco, Maria Francesca Bedeschi, Marco Venturin, Roberta Villa, Jole Costanza, Alessia Mauri, Carlo Santaniello, Odoardo Picciolini, Laura Messina, Fabio Triulzi, Monica Rosa Miozzo, Ornella Rondinone, Laura Fontana

**Affiliations:** 1 Medical Genetics, Department of Health Sciences, Università degli Studi di Milano, 20142 Milan, Italy; giada.moresco@unimi.it (G.M.); monica.miozzo@unimi.it (M.R.M.); laura.fontana@unimi.it (L.F.); 2 Medical Genetics Unit, Fondazione IRCCS Ca’ Granda Ospedale Maggiore Policlinico, 20122 Milan, Italy; mariafrancesca.bedeschi@policlinico.mi.it; 3 Department of Medical Biotechnology and Translational Medicine, Università degli Studi di Milano, 20054 Milan, Italy; marco.venturin@unimi.it; 4 Medical Genetics Unit, ASST Santi Paolo e Carlo, 20142 Milan, Italy; roberta.villa@asst-santipaolocarlo.it; 5 Research Laboratories Coordination Unit, Fondazione IRCCS Ca’ Granda Ospedale Maggiore Policlinico, 20122 Milan, Italy; jole.costanza@gmail.com (J.C.); alessia.mauri@unimi.it (A.M.); carlosantaniello8@gmail.com (C.S.); 6 Pediatric Physical Medicine & Rehabilitation Unit, Fondazione IRCCS Ca’ Granda Ospedale Maggiore Policlinico, 20122 Milan, Italy; odoardo.picciolini@policlinico.mi.it (O.P.); laura.messina@policlinico.mi.it (L.M.); 7 Neuroradiology Unit, Fondazione IRCCS Ca’ Granda Ospedale Maggiore Policlinico, 20122 Milan, Italy; fabio.triulzi@policlinico.mi.it

**Keywords:** Moebius syndrome, WES, rare disease, cohort analysis, trio analysis

## Abstract

Moebius syndrome (MBS) is a rare congenital disorder characterized by non-progressive facial palsy and ocular abduction paralysis. Most cases are sporadic, but also rare familial cases with autosomal dominant transmission and incomplete penetrance/variable expressivity have been described. The genetic etiology of MBS is still unclear: de novo pathogenic variants in *REV3L* and *PLXND1* are reported in only a minority of cases, suggesting the involvement of additional causative genes. With the aim to uncover the molecular causative defect and identify a potential genetic basis of this condition, we performed trio-WES on a cohort of 37 MBS and MBS-like patients. No de novo variants emerged in *REV3L* and *PLXND1*. We then proceeded with a cohort analysis to identify possible common causative genes among all patients and a trio-based analysis using an in silico panel of candidate genes. However, identified variants emerging from both approaches were considered unlikely to be causative of MBS, mainly due to the lack of clinical overlap. In conclusion, despite this large cohort, WES failed to identify mutations possibly associated with MBS, further supporting the heterogeneity of this syndrome, and suggesting the need for integrated omics approaches to identify the molecular causes underlying MBS development.

## 1. Introduction

Moebius or Möbius syndrome (MBS) is an extremely rare congenital neurological disorder with an estimated prevalence of 1/250,000 live births and equal incidence in both sexes. It is caused by the absence or underdevelopment (either unilaterally or bilaterally) of the VI and VII cranial nerves (CNs), leading to complete facial paralysis, with severe deficiency of food and speech function, as well as inability to move the eyes from side to side. MBS patients usually have normal intelligence, but in childhood they may have delayed speech because of the paralysis of muscles that move the lips, soft palate, and tongue root [1]. According to the diagnostic criteria defined in 2007 during the First Scientific Conference on Möbius Syndrome in Bethesda, Maryland, the minimum clinical diagnostic criteria for MBS are the following: a congenital, uni- or bilateral, non-progressive facial weakness with limited abduction of the eye(s) and full vertical motility. Patients who do not meet these criteria are labeled “Moebius-like” (MBS-like).

Abnormalities in the facial nerve (CN VII) and in the abducens nerve (CN VI) are the typical features, while hypoglossal nerve (CN XII) involvement is relatively common [2,3]. Occasionally, CN V and VIII are also affected [3], and hearing impairment is observed in patients with CN VIII dysfunction. Other symptoms sometimes associated with MBS are musculoskeletal deformities: these include clubfoot (which is the most common), brachydactyly, syndactyly, ectrodactyly, oligodactyly, acheiria, arthrogryposis, kyphosis, and scoliosis. Orthodontic issues can also be present in MBS patients, such as diminished temporo-mandibular movements, dental malocclusion with micrognathia, tongue malformation, and excessive maxillary development. In addition, MBS may be associated with chest-wall abnormalities (Poland sequence) and strabismus. Although rare, Poland sequence should be included in the differential diagnosis for a unilateral hyperlucent hemithorax on chest radiography, especially in the absence of cardiopulmonary symptoms [4]. Other differential diagnoses include Carey–Fineman–Ziter syndrome, Oro-Mandibular-Limb Hypogenesis syndrome (OMLH), Hypoglossia-hypodactyly syndrome, and Glossopalatine ankylosis.

The diagnosis of MBS is based exclusively on clinical criteria, although recent studies are beginning to document causative genetic patterns. MBS can be recognized and early diagnosed during the neonatal period with electromyographic examination and brain magnetic resonance imaging (MRI). Key clinical findings include poor or absent sucking due to incomplete closure of the lips, lack of facial mimicking (especially while crying), fixed gaze, incomplete eyelid closure during sleep, and ptosis. The difficulty of reaching a diagnosis and the variability/complexity of MBS require a multidisciplinary approach to establish a definite diagnosis and manage these children.

It has been suggested that the pathological mechanism underlying MBS involves rhombencephalon maldevelopment, predominantly affecting motor nuclei and axons, and traversing long tracts [2]. However, this theory has not been fully established yet.

Picciolini et al. suggested that MBS children exhibit not only primary deficits affecting movement, food, vision, and language, but also secondary developmental issues, such as visual exploration and oral-motor deficits, as well as difficulties in categorizing facial expressions, which affect cognitive strategies in early development. In addition, pain and other eating difficulties, maternal concerns, and lack of recognition of emotions can contribute to significant emotional distress [5].

It is estimated that there are 2 to 20 cases of MBS per million births, and although most cases of MBS are sporadic, familial occurrence has also been reported [2], suggesting a possible genetic basis. Autosomal dominant and autosomal recessive inheritance patterns were both proposed [6] and a family with possible X-linked recessive inheritance was described as well [7].

Both genetic and non-genetic factors are believed to play a role in MBS development, although the genetic cause remains largely unknown. The first genetic study on MBS suggested the presence of causative genes at the 13q12.2-q13 chromosomal region [8,9], but a further study ruled out microdeletions of the critical region and the role of three putative causative genes (*FGF9*, *GSH1,* and *CDX2*) as causative of MBS [10]. Two additional loci were identified in the 3q21-q22 and 10q21.3-q22 chromosomal regions in two large unrelated Dutch MBS pedigrees, revealing autosomal dominant inheritance [11,12]. However, mutation analysis of the candidate MBS genes located at these loci (*SOX14*, *PGT*, *GATA2,* and *PLXND1* on chromosome 3 and *EGR2* on chromosome 10) failed to identify mutations in MBS patients [13,14,15]. Mutations in the *RYR1* gene have been associated with atypical MBS or congenital fibrosis of the extraocular muscles (CFEOM), and the mutation c.1228G>A (p.Glu410Lys) in the *TUBB3* gene has been detected in a patient affected by congenital external ophthalmoplegia sparing abduction, facial weakness, anosmia, and hypogonadotropic hypogonadism, which overlaps with atypical MBS [16,17].

Moreover, cytogenetic anomalies have been reported in MBS-like patients, including the reciprocal translocations t(1;11)(p22;p13) [18] and t(1;2)(p22.3;q21.1) [19] and the paracentric inversion in the long arm of chromosome 8 (46,XX,inv(8)(q21.3q24.13)) [20].

In 2015, de novo mutations in the *PLXND1* and *REV3L* genes were identified by whole exome sequencing (WES) in two trios and six unrelated sporadic MBS patients [21]. The clinical significance of *PLXND1* and *REV3L* in the etiology of MBS is yet unknown. The two genes are involved in different pathways: *PLXND1* is involved in neural migration during hindbrain development, and *REV3L* plays a role in DNA translesion synthesis, a specific DNA repair process [21]. Knock-out mice for these two genes supported their causative role in MBS [21].

In addition, the complex chromosomal rearrangement 46,XY,t(7;8;11;13) was recently described in a patient with MBS [22]. Mapping of the chromothripsis breakpoints allowed for the identification of 12 truncated protein-coding genes, including *SEMA3A*, *SEMA3D, UBR5,* and *PIK3CG*, whose encoded proteins are known or predicted to interact with PLXND1 and REV3L. Moreover, variants in the *PLXND1* gene inherited from unaffected parents have been described as well [23], suggesting that either penetrance varies widely between cases or unidentified factors contribute to phenotypic expression in genetically predisposed individuals. Finally, the two novel genes *LMX1A* and *CHN1* have been recently proposed to be associated with MBS, as de novo missense variants were identified in two distinct MBS patients [24,25].

Despite numerous attempts to understand the etiology of MBS, mutations in the above-mentioned genes have been found in only a small percentage of patients or in isolated cases, mainly due to the difficulty in gathering large cohorts of MBS patients. This leaves the genetic origin of this disease largely unsolved. To shed further light on the molecular cause of MBS and identify a potential genetic basis of this condition, we recruited a large cohort of 37 probands in which we carried out trio-WES. Neither de novo mutations in the *PLXND1* and *REV3L* genes nor pathogenic mutations in other MBS candidate genes were found. We then extended the analysis using two approaches: a cohort analysis, in which we selected genes with rare and/or novel mutations present in at least two affected individuals, and a trio-WES analysis. However, no relevant variants strongly associated with the MBS phenotype emerged. To our knowledge, this is the largest MBS cohort analyzed by trio-WES to date, further supporting the heterogeneity of MBS and suggesting that additional factors, other than genetic mutations, likely play a crucial role in the development of this syndrome.

## 2. Materials and Methods

### 2.1. Patient Recruitment

MBS patients and their parents were recruited, between 27 January 2017 and 14 July 2021, at the Outpatient Center for the Clinical and Functional Diagnosis of Moebius Syndrome of Fondazione IRCCS Ca’ Granda Ospedale Maggiore Policlinico in Milan, Italy. Participants were also recruited through the Italian Moebius Syndrome Association OdV (AISMo OdV), the MBS Referral Center of the Hospital of Parma, and from individual pediatricians.

The clinic provides centralized evaluations of patients, primarily children, with confirmed or suspected clinical diagnosis of MBS. Patients were evaluated by a multidisciplinary team including geneticists, physiatrists, speech therapists, and psychologists. Each patient underwent a detailed phenotypic examination, including physical and functional evaluation, and a review of the medical records and of the family history up to the second degree. We gathered data on pregnancy and perinatal history, infant feeding, motor and language development, cognitive abilities, facial and physical features (including major and minor anomalies), and results from previous genetic tests (Table S1A,B).

The clinical diagnosis of MBS was established according to the major criteria outlined by the First Scientific Conference on Moebius Syndrome in 2007 [26]. The classic phenotype was defined by the presence of bilateral congenital VI and VII CN palsy. Children who exhibited additional CN involvement and/or motor, musculoskeletal, and neurodevelopmental disorders were also included in the study. Patients who did not meet both the major criteria were classified as MBS-like.

All participating families provided written informed consent to the study, which was approved by the local Ethics Committees (n. 549 of 26 February 2021).

### 2.2. WES and Bioinformatic Analysis

Genomic DNA was extracted from 400 μL of peripheral blood lymphocytes from all enrolled trios using the QIAsymphony DSP DNA Midi kit (QIAGEN, Hilden, Germany), according to the manufacturer’s instructions. Nanodrop (Thermo Fisher Scientific Inc., Waltham, MA, USA) and Tapestation (Agilent, Santa Clara, CA, USA) were used to assess DNA quantity, purity, and quality/integrity. Libraries were prepared using the SureSelectQXT Clinical Research Exome V2 kit (Agilent) and sequenced by 151 bp paired-end reads on the Illumina NextSeq 550 platform (Illumina, San Diego, CA, USA). Alignment, variant calling, annotation, and filtering were performed using previously described procedures [27].

Two different approaches were applied to identify potential MBS causative variants: cohort and a trio-based analyses. In the cohort analysis, we selected genes with rare and/or novel mutations present in at least two affected individuals and excluding those variants also found in healthy parents of other trios. Several in silico prediction tools, such as SIFT (v 6.2.1) [28], PolyPhen2 (v 2.2.3) [29], and MutationTaster (v 2021) [30], were used to evaluate the pathogenic score of identified variants. In addition, databases like HGMD (v 2023.3) [31], ClinVar [32], and OMIM [33] were interrogated to identify potential disease associations.

In the trio-based approach, the same VCF files were analyzed using the eVAI software (v 3.2) (enGenome, Pavia, Italy). An in silico panel of candidate genes (listed in Table S2) was assembled from the literature data and protein interaction databases.

All variants emerging from both approaches were interpreted according to the American College of Medical Genetics and Genomics (ACMG) guidelines [34] and using tools such as Varsome [35] and Franklin by Genoox (Palo Alto, CA, USA—94306) [36], thus allowing for the classification of identified variants in the standard five classes of pathogenicity (class 1–5). Variants classified as of uncertain significance (VUSs), likely pathogenic, or pathogenic underwent further literature review. Dominant inherited variants were considered only if classified as likely pathogenic or pathogenic.

Candidate mutations, with a possible correlation and/or strong in silico pathogenicity score that emerged from both the approaches, were verified by Sanger sequencing. Primers and protocols are available upon request.

Since RNA and protein samples were unavailable, functional studies were not performed to investigate the functional consequences of the identified variants. Instead, the MutationTaster tool [30] was used to establish the position of premature termination codons compared to the canonical ones and to predict the impact of nonsense-mediated decay, based on the rules governing this process [37].

## 3. Results

### 3.1. Clinical Phenotypes

We enrolled a total of 37 patients (17 males and 20 females) in our study, and among these, 30 are classified as MBS and 7 as MBS-like. Excluding the patient from Family 5, whose mother was Chinese, our cohort included 36 Caucasian subjects, primarily Italian, aged from 0 to 39 years (mean age: 7.9 years, median age: 6 years).

Table 1 summarizes the clinical details of these 37 probands, with further features provided in Table S1A,B (Supplementary Material). Of these, 30 presented both the characteristic features and were classified as MBS. Since six patients presented without impairment of the VI CN and one without involvement of the VII CN, they were classified as MBS-like. MBS was also associated with Poland sequence in two cases.

All cases were sporadic, with negative family histories of genetic disorders. Most of these MBS patients were born at term after an uneventful pregnancy, and no administration of Misoprostol or any other known teratogen was reported. Three cases were late preterm. Pregnancies were complicated by ultrasound evidence of bilateral or unilateral clubfoot in 12 cases, associated with intrauterine growth retardation in two cases, and associated with ventriculomegaly in one case.

Cerebral MRI was performed for 30 patients, of which 24 had normal results. Identified cerebral anomalies included brainstem hypoplasia (four cases), pons hypoplasia (two cases), and partial corpus callosum agenesis/thinning (four cases). The VII CN (total/partial, bilateral, or unilateral) was affected in 97% of cases, and the VI CN in 83%. Other CNs were altered in 40% of cases, especially the XII CNs. As a consequence of hypoplastic XII CNs, five patients affected showed hypoplastic tongue.

Other extra central nervous system malformations observed included clubfoot (32%, with six cases manifesting bilateral defects), hand anomalies, such as symbrachydactyly or fingers hypoplasia (13%), and thorax anomalies (19%). Minor facial anomalies observed included blepharophimosis, epicanthic folds, exotropia, hypertelorism, and micrognathia.

Neonatal hypotonia was relatively frequent in our cohort (19%). Poor neonatal sucking and feeding difficulties affected 51% of the patients, and three required nasogastric tubes or gastrostomy.

The most significant functional deficit, observed in half of the patients, was speech delay, including dyslalia and dysarthria. Moderate, mild, and severe intellectual disability was observed in 22% of patients, while the remaining showed normal intellectual abilities appropriate for their age.

Disturbances of the visual domain, including deficits in ocular motility and neurovisual function, affected 84% and 32% of children, respectively.

Finally, emotional and behavioral issues were present in 43% of cases, likely secondary to difficulties in social relationships with other people due to their genetic disease.

### 3.2. Genetic Analysis

WES identified an average of approximately 100,000 variants, with a mean average Q30 sequencing percentage of 88.4% and a uniform coverage exceeding 100×.

We first assessed the presence of variants in the *PLXND1* and *REV3L* genes, which are known to be causative of MBS [21]. We focused on de novo variants, given that our cohort was mainly characterized by sporadic cases, but no de novo mutations were identified in the *PLXND1* and *REV3L* genes. However, variants in these two genes inherited from unaffected parents were identified in 10 probands (Table 2). All inherited variants are classified as benign or likely benign, with the exception of two VUSs (c.3668A>C and c.4662G>T) in the *PLXND1* gene detected by analyzing Family 22 and Family 29, respectively, both inherited from the healthy fathers. Although these VUSs had not been previously described, they were also present in the unaffected parent, suggesting that they are unlikely to be disease-causing, unless incomplete penetrance is assumed.

We then proceeded with a cohort analysis approach to identify common causative genes across all probands enrolled in the study. We focused on genes with rare variants identified in at least two affected individuals. No de novo variants were found in genes shared by multiple patients but only variants inherited from an unaffected parent. The results of this analysis are summarized in Table 3.

The most significantly enriched gene was *RYR1*, in which three distinct heterozygous and likely pathogenic variants were identified in the patients of Family 10 (4293+2T>C), Family 30 (c.14761A>G, p.(Ile4926Val)), and Family 37 (c.7754C>T, p.(Thr2585Ile)), all inherited from the unaffected fathers (see Table 3). Mutations in this gene are associated with malignant hyperthermia susceptibility (AD, OMIM #145600), central core disease (AD, OMIM #117000), minicore myopathy with external ophthalmoplegia (AR; CMYP1B, OMIM #255320), and King-Denborough syndrome (AD, OMIM #619542). Shaaban et al. [16] reported two cases of atypical MBS, in which homozygous or compound heterozygous mutations in *RYR1* were identified, leading to the re-diagnosis of CMYP1B [16]. Since our patients carrying *RYR1* mutations do not show the typical signs of CMYP1B (e.g., ophthalmoplegia and severe hypotonia) and heterozygous *RYR1* mutations were inherited from the unaffected fathers, we suppose that these variants are unlikely to be causative of their clinical condition.

We also identified two missense variants, both classified as VUSs, in the *USP9X* gene in the two male probands of Family 12 (c.953C>T, p.(Ala318Val)) and Family 30 (c.3832G>C, p.(Asp1278His)). Mutations in this gene have been reported to cause X-linked intellectual disability with both recessive (OMIM #300919) and dominant female-restricted (OMIM #300968) inheritance. Both MBS patients inherited the *USP9X* mutations from their unaffected mothers. The identified missense variants occur in a protein portion where rare and pathogenic variants have not been reported. Moreover, patients carrying these *USP9X* mutations present with clinical features characteristic of MBS without intellectual disability, which is the main clinical feature of *USP9X* patients. Thus, we infer that the identified *USP9X* mutations do not cause MBS in these patients.

No other common mutated genes with relevant mutations were found in our cohort.

We then performed trio-based analyses for all MBS families using an in silico panel of candidate genes (Table S2) derived from the literature data and protein interaction databases. Relevant variants with their respective pathogenic scores are summarized in Table 3. Among all the trios, a novel de novo likely pathogenic nonsense variant in the *OPA1* gene (c.278T>G, (p.Leu93*)) was identified in the proband of Family 17. *OPA1* variants are known to cause the autosomal dominant optic atrophy (OPA1; OMIM #165500), which is characterized by progressive bilateral vision loss with onset during the first decade of life, central visual field defects, color vision disturbances, and optic disc pallor [38]. However, the lack of clinical overlap with MBS did not support a causative role for the identified *OPA1* mutation in the carrier patient.

In the proband of Family 14, WES analysis identified a novel de novo heterozygous variant in the *CHRNB1* gene (c.607A>G, p.(Ile203Val)), classified as a VUS. *CHRNB1* variants are known to cause congenital myasthenic syndrome (CMS2A; OMIM #616313, AD), characterized by abnormal fatigability and transient/permanent weakness of extraocular, facial, bulbar, truncal, respiratory, or limb muscles. While CMS2A can sometimes be misdiagnosed as MBS [39], our patient does not show any clinical feature of the congenital myasthenic syndrome, thus excluding this variant as causative of MBS.

Except for these two variants, which have been reported either for the pathogenicity or because they are responsible for a syndrome partially overlapping with MBS, we did not identify any other de novo or inherited variants that could be causative of MBS in this trio-based approach.

Finally, we reviewed the ACMG classification of the thirteen variants previously reported in the literature as causative of MBS and reclassified them according to the current ACMG guidelines (Table 4). One was classified as likely benign, one as likely benign/VUS, six as VUSs, three as likely pathogenic, and two as pathogenic.

## 4. Discussion and Conclusions

MBS is a rare congenital disorder with a complex, multifactorial etiology that includes vascular insufficiency, teratogens, infections, maternal or birth trauma, and genetics [41]. However, the genetic bases of MBS remain poorly understood and still need to be clarified.

In this study, we performed trio-WES in a cohort of 37 patients to uncover the genetic etiology of MBS. A key finding was the absence of de novo mutations in the *PLXND1* and the *REV3L* genes (Table 2), which were previously considered causative genes for MBS, according to studies involving animal models of the syndrome. Tomas-Roca et al. [21], indeed, demonstrated in mouse models that the knocking-out of these two genes independently leads to defects in the facial branchiomotor neuron migration and craniofacial bone abnormalities/vertebral defects, which are observed in MBS patients. Despite evidence supporting their involvement in MBS, these genes have not consistently shown a strong association in humans and are only associated with a minority of MBS cases [21].

Moreover, we also extended the analysis to inherited mutations in the *REV3L* and *PLXND1* genes, as variants in the *PLXND1* gene inherited from an unaffected parent have been described as well [23]. In our study, we identified inherited variants in the *REV3L* and *PLXND1* genes in three and eight patients, respectively (Table 2), but none were classified as pathogenic or likely pathogenic according to ACMG criteria, thus, we excluded their potential contribution to the condition.

In line with previous WES studies of MBS patients [40,42,43], we failed to find mutations in these two genes, thus suggesting the necessity to reevaluate their significance in MBS and explore other potential loci that may cause the syndrome.

Given the exceptional number of recruited patients, we performed a cohort analysis to identify possible causative mutations present in at least two MBS patients. We identified shared mutations in the *RYR1* and the *USP9X* genes. In particular, we found three distinct heterozygous and likely pathogenic variants in the *RYR1* gene in three MBS patients (Family 10, 30, and 37). This gene encodes the ryanodine receptor 1, which functions both as a calcium release channel in the sarcoplasmic reticulum and as a linker between the sarcoplasmic reticulum and transverse tubule [44]. *RYR1* variants are associated with several conditions, including CMYP1B [45]. However, the mutations identified in our cohort were inherited from unaffected fathers, and the clinical presentation of these MBS patients did not overlap with CMYP1B [45], ruling out a causative role of these variants in MBS development.

We also identified mutations in the X-linked *USP9X* gene, which encodes a substrate-specific deubiquitylating enzyme involved in dendritic spine maturation and synaptic plasticity [46]. This gene was mutated in two unrelated male patients (Family 12 and 30). However, these variants are unlikely to be causative given the lack of a correlation with the clinical features typically associated with *USP9X* mutations.

Overall, our results from the cohort analysis suggest a limited role for genetics in the development of MBS, which was further supported by the trio-WES analysis. The only variants identified were two heterozygous variants in the *OPA1* and the *CHRNB1* genes. In particular, the *OPA1* gene encodes a dynamin-related GTPase that is located in the inner mitochondrial membrane and helps the regulation of mitochondrial fusion and fission [47], and the *CHRNB1* gene encodes the β subunit of the acetylcholine receptor at the neuromuscular junction [39]. However, these genes are associated with conditions (OPA1 and CMS2A, respectively) whose clinical features do not overlap with the phenotypes observed in the patients of our cohort.

Finally, we reviewed the ACMG classification of the 13 variants previously reported in the literature as causative of MBS and reclassified them according to the current ACMG guidelines (Table 4). Most MBS variants are now classified as a VUS or LP, which raises questions about their pathogenicity and warrants a further revision of their role in MBS development. Moreover, this reclassification highlights the importance of performing a more comprehensive mutational analysis, without focusing only on those genes already associated with the condition.

In conclusion, since we performed trio-WES on the largest MBS cohort to our knowledge without revealing mutations clearly associated with MBS development, this study further supports the heterogeneous nature of the syndrome and suggests that both genetic and non-genetic factors may be involved. Nevertheless, despite WES representing an effective approach for undiagnosed rare diseases, it might miss different types of genomic variation (e.g., repeat expansions and deep intronic variants). The combination of different omics approaches, in addition to environmental risk factors, maternal exposures, and potential gene–environment interactions, may shed light on the pathomechanisms of MBS development.

## Figures and Tables

**Table 1:** Clinical features reported in MBS and MBS-like patients of our cohort, with the corresponding number and percentage of patients in which each feature was observed.

Clinical Feature	Number of Patients
	(% on the Total)
Gender	
Females	20 (54)
Males	17 (46)
Specific Cranial Nerve Involvement	
Abducens Nerve (VI) palsy	31 (83)
Facial Nerve (VII) palsy	36 (97)
Neurological	
Other CN palsy	15 (40)
Brain imaging anomalies	6/30 † (20)
Neonatal hypotonia	7 (19)
Motor development delay	4 (11)
Motor impairment	7 (19)
Language development delay	8 (22)
Speech deficit	19 (51)
Intellectual disability	8 (22)
Behavioral problems	15/35 ‡ (43)
Oculomotor motility deficit	31 (84)
Neurovisual deficit	12 (32)
Hearing deficit	4 (11)
Craniofacial	
Facial dysmorphisms	6 (16)
Esotropia/Exotropia	28/31 § (90)
Palate anomalies	6 (16)
Hypoplastic tongue	5 (13)
Micrognathia	7 (19)
Musculo-skeletal	
Thorax Anomalies	7 (19)
Upper Extremity Anomalies	5 (13)
Lower Extremity Anomalies	15 (41)
Respiratory	
Respiratory Difficulties in infancy	5 (13)
Cardiovascular	4 (11)
Gastrointestinal	
Feeding problems in infancy	19 (51)
Genito-urinary	1 (3)

† The total number of patients that underwent cerebral MRI is 31. ‡ We evaluated behavioral problems starting from patients aged 2. § We evaluated esotropia/exotropia only in those patients presenting with VI CN deficit.

**Table 2:** Heterozygous variants identified in the *PLXND1* (NM_015103.2) and *REV3L* (NM_001286432.1) genes in the probands of our cohort.

Family	Gene	Variant	Parental Origin	ACMG Classification
3	*PLXND1*	3′UTR	Paternal	LB
		c.*1088G>A		
4	*REV3L*	Missense	Paternal	LB/VUS
		c.3153G>T		
		p.Trp1051Cys		
7	*PLXND1*	3′UTR	Paternal	B
		c.*841C>G		
14	*PLXND1*	Missense	Paternal	B
		c.3505C>T		
		p.Arg1169Cys		
	*PLXND1*	Missense	Maternal	B
		c.2275C>T		
		p.Pro659Ser		
22	*PLXND1*	Missense	Paternal	VUS
		c.3668A>C		
		p.Asp1223Ala		
27	*PLXND1*	Missense	Paternal	LB
		c.3448G>T		
		p.Val1150Leu		
29	*PLXND1*	Missense	Paternal	VUS
		c.4662G>T		
		p.Lys1554Asn		
32	*PLXND1*	Missense	Maternal	LB
		c.5671G>A		
		p.Ala1891Thr		
33	*PLXND1*	Missense	Paternal	B
		c.1501G>A		
		p.Glu501Lys		

**Table 3:** Details of the variants identified by the cohort-WES and trio-WES analyses in our cohort of MBS and MBS-like patients.

	Family	Gene (Refseq)	Variant	Allelic State	Inheritance	ACMG Classification	OMIM Data
Cohort-WES analysis	10	*RYR1* (NM_000540.3)	Splicing	Heterozygous	Paternal	LP	Malignant hyperthermia susceptibility 1, AD (MHS1; #145600); Congenital myopathy 1A with susceptibility to malignant hyperthermia, AD (CMYP1A; #117000); Congenital myopathy 1B, AR (CMYP1B; #255320); King-Denborough syndrome, AD (KDS; #619542)
			c.4293+2T>C				
			No rs				
	30		Missense	Heterozygous	Paternal	LP	
			c.14761A>G				
			p.Ile4926Val				
			rs746765251				
	37		Missense	Heterozygous	Paternal	VUS/LP	
			c.7754C>T				
			p.Thr2585Ile				
			rs371934483				
	12	*USP9X* (NM_001039591)	Missense	Hemizygous	Maternal	VUS/LP	Intellectual developmental disorder X-linked 99 (XLID99; #300919), XLR
			c.953C>T				
			p.Ala318Val				
			No rs				
	30		Missense	Hemizygous	Maternal	VUS/LP	
			c.3832G>C				
			p.Asp1278His				
			rs980783156				
Trio-WES analysis	17	*OPA1 (NM_130836)*	Nonsense	Heterozygous	De novo	P	Optic atrophy with or without deafness, ophthalmoplegia, myopathy, ataxia, and neuropathy (OPA1; #125250), AD
			c.278T>G				
			p.(Leu93Ter)				
			No rs				
	14	*CHRNB1 (NM_000747)*	Missense	Heterozygous	De novo	VUS	Myasthenic syndrome, congenital, 2A, slow-channel (CMS2A; #616313), AD

**Table 4:** Revision of the ACMG classification of variants reported in the literature to be causative of MBS or MBS-like phenotypes.

Gene	Transcript and Variant	Phenotype	Parental Origin (Inheritance)	ACMG Classification	Reference
*RYR1*	NM_000540	atypical MBS/CFEOM	Both (AR)	VUS	[16]
	c.2966A>G, p.Glu989Gly				
*RYR1*	NM_000540	atypical MBS/CFEOM	Both/paternal (AR/compound het)	LP	[16]
	c.11314C>T, p.Arg3772Trp				
*RYR1*	NM_000540	atypical MBS/CFEOM	Maternal (compound het)	P	[16]
	c.848A>G, p.His283Arg				
*PLXND1*	NM_015103	MBS	De novo (AD)	VUS	[21]
	c.5685C>A, p.Asn1895Lys				
*PLXND1*	NM_015103	MBS	De novo (AD)	VUS	[21]
	c.4454_4455GC>CA, p.Arg1485Pro				
*PLXND1*	NM_015103	MBS	De novo (AD)	LB	[21]
	c.3018C>T, p.Leu1006Leu				
*REV3L*	NM_002912	MBS	De novo (AD)	LP	[21]
	c.1096+1G>A				
*REV3L*	NM_002912	MBS	De novo (AD)	LB/VUS	[21]
	c.1160A>G, p.Glu387Gly				
*REV3L*	NM_002912	MBS	De novo (AD)	LP	[21]
	c.2662A>T, p.Lys888*				
*CCDC160*	NM_001101357	MBS	De novo (AD)	VUS	[21]
	c.501delA, p.Glu167Aspfs*21				
*TUBB3*	NM_006086.3	atypical MBS/CFEOM	De novo (AD)	LP/P	[40]
	c.1228G>A, p.Glu410Lys				
*PLXND1*	NM_015103.3	Poland-Moebius Syndrome	Maternal (AD †)	VUS	[23]
	c.2890G>A, p.Val964Met				
*LMX1A*	NM_177398.4	MBS	De novo (AD)	VUS	[24]
	c.182A>G, p.Gln61Arg				
*CHN1*	NM_001822.7	MBS	De novo (AD)	LP	[25]
	c.643G>A, p.Gly215Arg				

AR: autosomal recessive; AD: autosomal dominant; AD †: autosomal dominant with supposed incomplete penetrance; het: heterozygous; CFEOM: congenital fibrosis of extraocular muscles, with or without extraocular involvement; HCFP3: hereditary congenital facial paresis.

## Data Availability

The data that support the findings of this study are available from the corresponding author, Ornella Rondinone, upon reasonable request.

## Supplementary Materials

The following supporting information can be downloaded at: https://www.mdpi.com/article/10.3390/genes15080971/s1, Table S1A: Neurofunctional features of our cohort; Table S1B: Clinical features of our cohort; Table S2: In-silico panel of candidate genes extracted from literature data and protein interaction databases.
